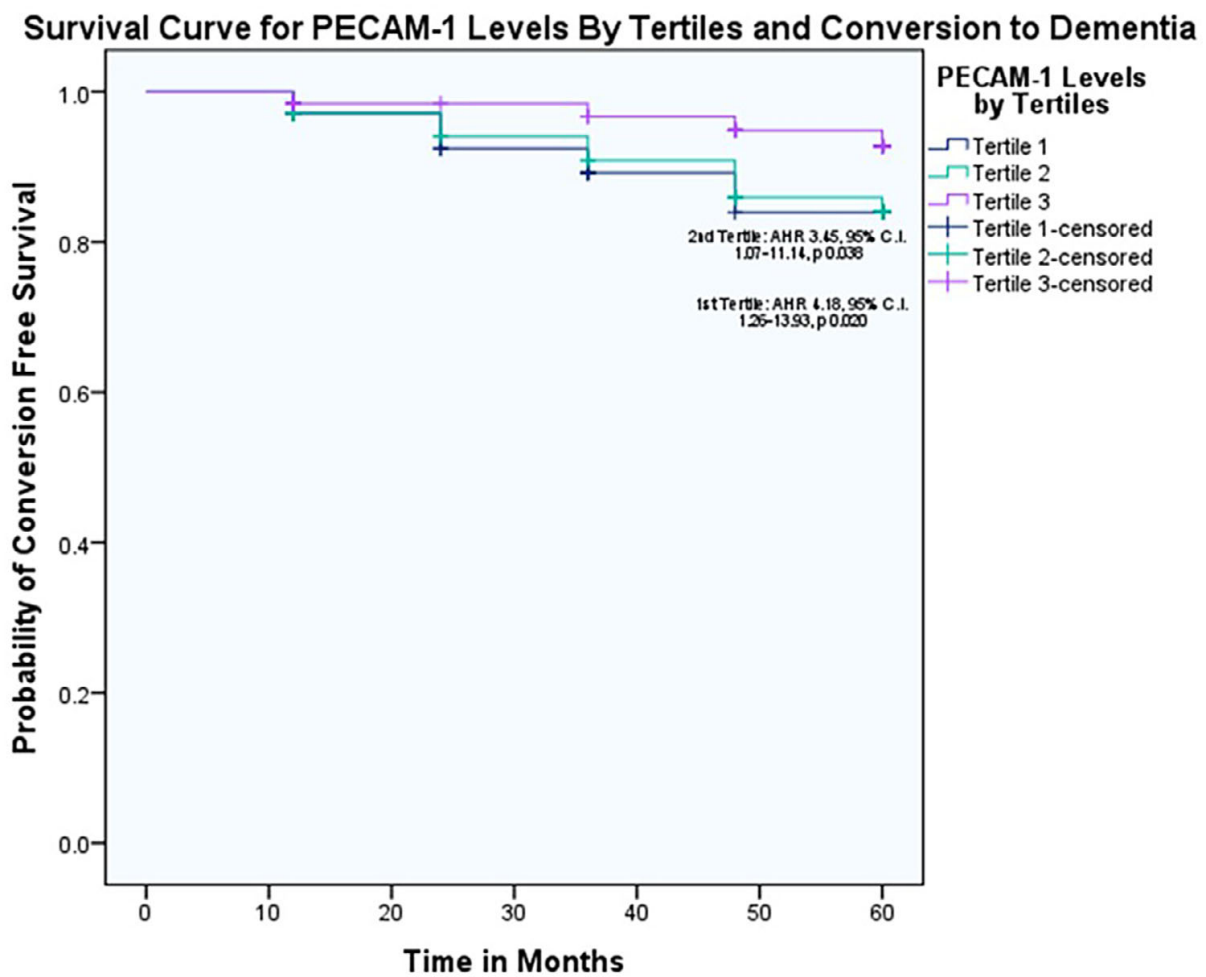# Associations of Circulating Platelet Endothelial Cell Adhesion Molecule‐1 Levels with Progression of Cerebral Small Vessel Disease, Cognitive Performance and Incident Dementia

**DOI:** 10.1002/alz.088139

**Published:** 2025-01-09

**Authors:** Ming Ann Sim, Eugene Tan, Siew‐Pang Chan, Yuek Ling Chai, Joyce R Chong, Narayanaswamy Venketasubramanian, Boon Yeow Tan, Mitchell Kim Peng Lai, Saima Hilal, Christopher Chen

**Affiliations:** ^1^ National University Hospital, Singapore, Singapore Singapore; ^2^ National University of Singapore, Singapore, singapore Singapore; ^3^ National University Heart Centre Singapore, Singapore, SIngapore Singapore; ^4^ National University Heart Centre Singapore, Singapore Singapore; ^5^ National University of Singapore, Singapore Singapore; ^6^ Yong Loo Lin School of Medicine, National University of Singapore, Singapore Singapore; ^7^ Memory Aging & Cognition Centre, National University Health System, Singapore Singapore; ^8^ Memory Aging and Cognition Center, National University Health System, Singapore Singapore; ^9^ Raffles Neuroscience Centre, Raffles Hospital, Singapore Singapore; ^10^ St. Luke's Hospital, Singapore Singapore; ^11^ National University of Singapore, Kent Ridge Singapore; ^12^ Saw Swee Hock School of Public Health, National University of Singapore and National University Health System, Singapore, Singapore Singapore

## Abstract

**Background:**

The association between platelet endothelial cell adhesion molecule‐1 (PECAM‐1) with cerebral small vessel disease (CSVD) and cognition in non‐demented subjects remains un‐investigated.

**Method:**

A longitudinal, prospective cohort of subjects recruited from memory clinics was followed‐up for 5 years. Non‐demented subjects were included and classified as no cognitive impairment (NCI) or mild cognitive impairment (MCI). Annual neuropsychological assessments and 2‐yearly magnetic resonance imaging (MRI) scans were performed. The associations of baseline circulating plasma PECAM‐1 levels with neuroimaging markers of CSVD, cognitive decline (Montreal Cognitive Assessment [MoCA] scores and executive function Z‐scores), and conversion to dementia were evaluated.

**Result:**

Of 213 subjects (age 70.2±7.7 years, 51.2% male, 122 (57.3%) NCI, and 91 (42.7%) MCI), median PECAM‐1 levels were 0.790 [IQR 0.645‐0.955] ng/ml). Compared with the highest tertile, subjects within the lowest PECAM‐1 tertile had higher age‐related white matter changes scores (first tertile: β 1.50, 95% C.I. 0.23‐2.77, p=0.02) and cerebral microbleeds (first tertile: Adjusted risk ratio [ARR] 2.59, 95% C.I. 1.81‐3.72, p<0.0001). PECAM‐1 levels were not associated with baseline MOCA and executive function.

Of the 204 participants with follow‐up data (median 60.0 [IQR 60.0‐60.0] months), 24 (11.8) had incident dementia. Compared with the highest tertile, subjects within the lower tertiles of PECAM‐1 were independently associated with higher risk of incident dementia (first tertile: Adjusted Hazard Ratio [AHR] 4.18, 95% C.I. 1.26‐13.93, p=0.020; second tertile: AHR 3.45, 95% C.I. 1.07‐11.14, p=0.038, Figure 1). The lowest PECAM‐1 tertile was associated with greater 4‐year progression of cerebral microbleeds (Incident Relative Risk [IRR] 2.44, 95% C.I. 1.20‐4.98, p=0.014), and decline in executive function (β ‐0.43, 95% C.I. ‐0.73, ‐0.14, p=0.004), and MoCA (β ‐1.32, 95% C.I. ‐2.30, ‐0.35, p=0.008) scores.

**Conclusion:**

In non‐demented subjects, lower circulating PECAM‐1 levels are associated with greater CSVD progression, cognitive decline, and incident dementia. PECAM‐1 may be a potential therapeutic target for CSVD and cognitive decline.